# Supplementary material for: Postoperative swallowing recovery in oral and oropharyngeal cancer: A prospective analysis of functional changes and adjuvant therapy effects
Source: PLoS One. 2026 Jan 27;21(1):e0341116. doi: 10.1371/journal.pone.0341116 (PMC12843576; doi:10.1371/journal.pone.0341116)
Supplement: S1 File — (ZIP) [file pone.0341116.s001.zip › Data Dictionary - EN.docx]

Data Dictionary

| **Variable name** | **Variable values** | **Variable label** |
| --- | --- | --- |
| no | Numeric (N = 89; from: 1 to 89) | Number |
| tuoi | Numeric (N = 89; from: 22 to 77) | Age |
| nhomtuoi | 0 = <65; 1 = ≥65 | Age group |
| gioi | 1 = Male; 2 = Female | Gender |
| bmi | Numeric (N = 89; from: 16.23 to 29.76) | Body Mass Index (BMI) |
| nhombmi | 1 = <18.5; 2 = 18.5–<23; 3 = ≥23 | BMI category |
| benhkem | 0 = No; 1 = Yes | Comorbidity |
| phanloaibenhkem | 0 = No comorbidity; 1 = Respiratory diseases (asthma, COPD, old pulmonary TB, newly diagnosed pulmonary TB, pneumonia); 2 = Diabetes mellitus | Comorbidity classification |
| phanloaik | 1 = Oral cavity; 2 = Oropharynx | Cancer classification by anatomical site |
| vitriungthu | 1 = Tongue – floor of mouth cancer; 2 = Base of tongue cancer; 3 = Tonsil cancer; 4 = Soft palate cancer; 5 = Posterior pharyngeal wall cancer | Primary tumor site |
| vitriungthugp | 0 = Oropharynx; 1 = Tongue – floor of mouth | Primary tumor site (grouped) |
| tumor | 1 = T1 stage; 2 = T2 stage; 3 = T3 stage; 4 = T4 stage | Tumor T stage |
| utientrien | 1 = Early-stage tumor; 2 = Advanced-stage tumor | Tumor progression stage |
| node | 0 = N0 stage; 1 = N1 stage; 2 = N2 stage; 3 = N3 stage | Cervical nodal stage |
| nodegp | 0 = N0; 1 = N1–N3 | Cervical nodal stage (grouped) |
| naohach | 0 = No neck dissection; 1 = Ipsilateral neck dissection; 2 = Bilateral neck dissection | Neck dissection at surgery |
| nhomhach | 0 = N/A; 1 = Levels 1–3; 2 = Levels 1–5 | Neck dissection levels |
| w_0 | Numeric (N = 89; from: 39 to 85) | Preoperative weight |
| m_0 | 3 = Can eat any food if prepared appropriately; 4 = Can swallow most foods but must be careful to avoid aspiration; 5 = Can eat all types of food | Preoperative feeding method |
| t_0 | 2 = 35–45 minutes; 3 = 25–35 minutes; 4 = 15–25 minutes; 5 = <15 minutes | Average mealtime before surgery |
| f_0 | 3 = Can eat porridge; 4 = Can eat soft foods like soft rice, soft vegetables; 5 = Can eat any type of food | Preoperative food consistency able to eat |
| eat_0 | Numeric (0–12) | Preoperative EAT-10 score |
| eat_gp_0 | 0 = No; 1 = Yes | Preoperative EAT-10 abnormality |
| kh_lsm_kieucat | 0 = None; 1 = Partial glossectomy; 2 = Type 3 glossectomy; 4 = Total mobile tongue resection; 5 = Resection of tongue ventral surface | Type of mobile tongue resection |
| kh_lsm_cattronkhoang | 0 = No; 1 = Subtotal compartment; 2 = Total compartment | Tongue–floor of mouth compartment resection |
| kh_lsm_kieucattronkhoang | 0 = No total compartment resection; 1 = Unilateral with tip preservation; 2 = Unilateral without tip preservation (one side); 3 = Unilateral without tip preservation (both sides); 4 = Bilateral with epithelial & intrinsic muscle preservation; 5 = Bilateral near-total or total | Type of total compartment tongue–floor of mouth resection |
| kh_lsm_taitao | 0 = No defect; 1 = Reconstruction without flap; 2 = Reconstruction with flap | Reconstruction method for tongue–floor of mouth defect |
| kh_dayluoi | 0 = None; 1 = Mucosal resection of tongue base; 2 = Unilateral tongue base resection; 3 = Bilateral tongue base resection | Tongue base defect |
| kh_kcm | 0 = None; 1 = Resection of < half of soft palate; 2 = Resection of > half but not total | Soft palate defect |
| kh_cxh_mui | 0 = No; 1 = Yes | Nasopharyngeal constrictor muscle defect |
| kh_cxh_mieng | 0 = No; 1 = Yes | Oropharyngeal constrictor muscle defect |
| kh_cxh_thanhquan | 0 = No; 1 = Yes | Laryngopharyngeal constrictor muscle defect |
| kh_cxh_2tang | 0 = No; 1 = Yes | ≥2-level pharyngeal constrictor defect |
| kh_cxh_taitao | 0 = No constrictor muscle defect; 1 = Reconstruction without flap; 2 = Reconstruction with flap | Reconstruction method for constrictor muscle defect |
| kh_loaivat | 0 = None; 1 = Submental flap; 2 = Supraclavicular fasciocutaneous flap; 3 = Pectoralis major flap; 4 = Other | Flap type for oropharyngeal reconstruction |
| cord_hm | 0 = No defect; 1 = No adverse feature; 2 = Adverse feature present | CORD classification for oropharyngeal defect |
| nhiemtrung_hp | 0 = No; 1 = Yes | Postoperative oral–pharyngeal infection |
| dieutribosung | 0 = None; 1 = Adjuvant radiotherapy; 2 = Adjuvant chemoradiotherapy | Adjuvant therapy |
| ngay_tapan | Numeric (3–28) | Postoperative day to start oral feeding |
| ngay_tapan_gp | 1 = ≤7; 2 = 8–14; 3 = >14 | Grouped postoperative day to start oral feeding |
| ngay_anhoantoan | Numeric (3–150) | Postoperative day to achieve full oral feeding |
| ngay_anhoantoan_gp | 1 = ≤7; 2 = 8–14; 3 = >14 | Grouped postoperative day to achieve full oral feeding |
| ns_daythanh_hp | 0 = Normal vocal cord movement; 1 = Weakness or unilateral vocal cord paralysis | Vocal cord status at the start of oral feeding |
| ns_udongdich_hp | 0 = None or a few secretion bubbles not bilateral and not deeply pooled; 1 = Deep pooling or bilateral pooling; 2 = Pooling in laryngeal vestibule disappearing after swallow or cough | Pharyngeal secretion retention at the start of oral feeding |
| ns_tinhchatdich_hp | 0 = No retained secretions; 1 = Bubbles; 2 = Mucus; 3 = Bubbles and mucus | Characteristics of retained pharyngeal secretions at the start of oral feeding |
| ns_dv_slnt_hp | 1 = Once; 2 = Multiple times | Number of additional swallows with moderately thick liquid at the start of oral feeding |
| ns_dv_hm_hp | 0 = None; 1 = Coating on mucosa; 2 = Mild residue; 3 = Deep residue | Moderately thick liquid – oropharyngeal residue at the start of oral feeding |
| ns_dv_hh_hp | 0 = None; 1 = Coating on mucosa; 2 = Mild residue; 3 = Deep residue | Moderately thick liquid – hypopharyngeal residue at the start of oral feeding |
| ns_dv_naptt_hp | 0 = None; 1 = Present; 2 = Grade 2 | Moderately thick liquid – epiglottic residue at the start of oral feeding |
| ns_dv_tiendinh_hp | 0 = None; 1 = Present; 2 = Grade 2 | Moderately thick liquid – laryngeal vestibule residue at the start of oral feeding |
| ns_dv_daythanh_hp | 0 = None; 1 = Present; 2 = Grade 2 | Moderately thick liquid – vocal fold residue at the start of oral feeding |
| ns_dv_hatm_hp | 0 = None; 1 = Present | Moderately thick liquid – subglottic residue at the start of oral feeding |
| ns_dv_lenmui_hp | 0 = No; 1 = Yes | Moderately thick liquid – nasal regurgitation at the start of oral feeding |
| ns_dv_km_hp | 0 = None; 1 = Coating on mucosa; 2 = Mild residue; 3 = Deep residue | Moderately thick liquid – oral cavity residue at the start of oral feeding |
| ns_dv_hitsac_hp | 0 = No; 1 = Yes | Moderately thick liquid – penetration or aspiration at the start of oral feeding |
| ns_dv_npn_hp | 0 = No; 1 = Yes | Moderately thick liquid – use of swallowing maneuver at the start of oral feeding |
| ns_dv_tennpn_hp | Text | Moderately thick liquid – name of effective swallowing maneuver |
| ns_dac_slnt_hp | 1 = Once; 2 = Multiple times | Number of additional swallows with extremely thick liquid at the start of oral feeding |
| ns_dac_hm_hp | 0 = None; 1 = Coating on mucosa; 2 = Mild residue; 3 = Deep residue | Extremely thick liquid – oropharyngeal residue at the start of oral feeding |
| ns_dac_hh_hp | 0 = None; 1 = Coating on mucosa; 2 = Mild residue; 3 = Deep residue | Extremely thick liquid – hypopharyngeal residue at the start of oral feeding |
| ns_dac_naptt_hp | 0 = None; 1 = Present; 2 = Grade 2; 3 = Grade 3 | Extremely thick liquid – epiglottic residue at the start of oral feeding |
| ns_dac_tiendinh_hp | 0 = None; 1 = Present; 2 = Grade 2; 3 = Grade 3 | Extremely thick liquid – laryngeal vestibule residue at the start of oral feeding |
| ns_dac_daythanh_hp | 0 = None; 1 = Present; 2 = Grade 2 | Extremely thick liquid – vocal fold residue at the start of oral feeding |
| ns_dac_hatm_hp | 0 = None; 1 = Present; 2 = Grade 2 | Extremely thick liquid – subglottic residue at the start of oral feeding |
| ns_dac_lenmui_hp | 0 = No; 1 = Yes | Extremely thick liquid – nasal regurgitation at the start of oral feeding |
| ns_dac_km_hp | 0 = None; 1 = Coating on mucosa; 2 = Mild residue; 3 = Deep residue | Extremely thick liquid – oral cavity residue at the start of oral feeding |
| ns_dac_hitsac_hp | 0 = No; 1 = Yes | Extremely thick liquid – penetration or aspiration at the start of oral feeding |
| ns_dac_npn_hp | 0 = No; 1 = Yes | Extremely thick liquid – use of swallowing maneuver at the start of oral feeding |
| ns_dac_tennpn_hp | Text | Extremely thick liquid – name of effective swallowing maneuver |
| ns_lg_slnt_hp | 1 = Once; 2 = Multiple times | Number of additional swallows with thin liquid at the start of oral feeding |
| ns_lg_hm_hp | 1 = Coating on mucosa; 2 = Mild residue | Thin liquid – oropharyngeal residue at the start of oral feeding |
| ns_lg_hh_hp | 0 = None; 1 = Coating on mucosa; 2 = Mild residue; 3 = Deep residue | Thin liquid – hypopharyngeal residue at the start of oral feeding |
| ns_lg_naptt_hp | 0 = None; 1 = Present; 2 = Grade 2 | Thin liquid – epiglottic residue at the start of oral feeding |
| ns_lg_tiendinh_hp | 0 = None; 1 = Present; 2 = Grade 2 | Thin liquid – laryngeal vestibule residue at the start of oral feeding |
| ns_lg_daythanh_hp | 0 = None; 1 = Present; 2 = Grade 2 | Thin liquid – vocal fold residue at the start of oral feeding |
| ns_lg_hatm_hp | 0 = None; 1 = Present; 2 = Grade 2 | Thin liquid – subglottic residue at the start of oral feeding |
| ns_lg_lenmui_hp | 0 = No; 1 = Yes | Thin liquid – nasal regurgitation at the start of oral feeding |
| ns_lg_km_hp | 0 = None; 1 = Coating on mucosa; 2 = Mild residue; 4 = Grade 4 | Thin liquid – oral cavity residue at the start of oral feeding |
| ns_lg_hitsac_hp | 0 = No; 1 = Yes | Thin liquid – penetration or aspiration at the start of oral feeding |
| ns_lg_npn_hp | 0 = No maneuver used; 1 = Maneuver used | Thin liquid – use of swallowing maneuver at the start of oral feeding |
| ns_lg_tennpn_hp | Text | Thin liquid – name of effective swallowing maneuver |
| sudungnpn_hp | 0 = No; 1 = Yes | Use of swallowing maneuver at the start of oral feeding |
| fois_hp | 0, 3, 4, 5, 6 | FOIS score at the start of oral feeding |
| w_1 | Numeric (N = 89; 33–74) | Weight at 1 month |
| m_1 | 2 = Mainly tube feeding with small oral intake; 3 = Can eat any food if appropriately prepared; 4 = Can swallow most foods but must be careful to avoid aspiration; 5 = Can eat all types of food | Feeding method at 1 month |
| t_1 | 2 = 35–45 min; 3 = 25–35 min; 4 = 15–25 min; 5 = <15 min | Average mealtime at 1 month |
| f_1 | 2 = Can swallow viscous liquids; 3 = Can eat porridge; 4 = Can eat soft foods (soft rice, soft vegetables); 5 = Can eat any type of food | Food consistency able to eat at 1 month |
| eat_1 | Numeric (0–30) | EAT-10 score at 1 month |
| eat_gp_1 | 0 = No; 1 = Yes | EAT-10 abnormality at 1 month |
| eat_cau1_1 | 0–4 | Dysphagia causes weight loss – 1 month |
| eat_cau2_1 | 0–4 | Dysphagia prevents eating comfortably in public – 1 month |
| eat_cau3_1 | 0–3 | Effort needed to swallow liquids – 1 month |
| eat_cau4_1 | 0–4 | Effort needed to swallow solids – 1 month |
| eat_cau5_1 | 0–4 | Effort needed to swallow pills – 1 month |
| eat_cau6_1 | 0–2 | Swallowing is very painful – 1 month |
| eat_cau7_1 | 0–4 | Dysphagia reduces enjoyment of eating – 1 month |
| eat_cau8_1 | 0–3 | Food sticks in throat – 1 month |
| eat_cau9_1 | 0–3 | Cough when eating/drinking – 1 month |
| eat_cau10_1 | 0–2 | Fear of swallowing – 1 month |
| ns_daythanh_1 | 0 = Normal vocal cord movement; 1 = Weakness or unilateral vocal cord paralysis | Vocal cord status at 1 month |
| ns_udongdich_1 | 0 = None or a few secretion bubbles not bilateral and not deeply pooled; 1 = Deep pooling or bilateral pooling; 2 = Pooling in laryngeal vestibule disappearing after swallow or cough; 3 = Pooling in laryngeal vestibule not disappearing after swallow or cough | Pharyngeal secretion retention at 1 month |
| ns_tinhchatdich_1 | 0 = No retained secretions; 1 = Bubbles; 2 = Mucus; 3 = Bubbles and mucus | Characteristics of retained pharyngeal secretions at 1 month |
| ns_dv_slnt_1 | 0 = No; 1 = Once; 2 = Multiple times | Number of additional swallows with moderately thick liquid at 1 month |
| ns_dv_hm_1 | 0 = None; 1 = Adhered to mucosa as streaks; 2 = Shallow residue; 3 = Deep residue | Moderately thick liquid – Oral cavity residue at 1 month |
| ns_dv_hh_1 | 0 = None; 1 = Adhered to mucosa as streaks; 2 = Shallow residue | Moderately thick liquid – Hypopharyngeal residue at 1 month |
| ns_dv_naptt_1 | 0 = None; 1 = Present; 2 = Score 2 | Moderately thick liquid – Epiglottic residue at 1 month |
| ns_dv_tiendinh_1 | 0 = None; 1 = Present; 2 = Score 2 | Moderately thick liquid – Laryngeal vestibule residue at 1 month |
| ns_dv_daythanh_1 | 0 = None; 1 = Present; 2 = Score 2 | Moderately thick liquid – Vocal fold residue at 1 month |
| ns_dv_hatm_1 | 0 = None | Moderately thick liquid – Subglottic residue at 1 month |
| ns_dv_lenmui_1 | 0 = No; 1 = Yes | Moderately thick liquid – Nasal regurgitation at 1 month |
| ns_dv_km_1 | 0 = None; 1 = Adhered to mucosa as streaks; 2 = Shallow residue | Moderately thick liquid – Oral cavity residue at 1 month |
| ns_dv_hitsac_1 | 0 = No; 1 = Yes | Moderately thick liquid – Penetration or aspiration at 1 month |
| ns_dv_npn_1 | 0 = No; 1 = Yes | Moderately thick liquid – Use of swallowing maneuver at 1 month |
| ns_dv_tennpn_1 | Text | Moderately thick liquid – Name of effective swallowing maneuver used at 1 month |
| ns_dac_slnt_1 | 0 = No; 1 = Once; 2 = Multiple times | Number of additional swallows with thick liquid at 1 month |
| ns_dac_hm_1 | 0 = None; 1 = Adhered to mucosa as streaks; 2 = Shallow residue; 3 = Deep residue | Thick liquid – Oral cavity residue at 1 month |
| ns_dac_hh_1 | 0 = None; 1 = Adhered to mucosa as streaks; 2 = Shallow residue; 3 = Deep residue | Thick liquid – Hypopharyngeal residue at 1 month |
| ns_dac_naptt_1 | 0 = None; 1 = Present; 2 = Score 2 | Thick liquid – Epiglottic residue at 1 month |
| ns_dac_tiendinh_1 | 0 = None; 1 = Present; 2 = Score 2 | Thick liquid – Laryngeal vestibule residue at 1 month |
| ns_dac_daythanh_1 | 0 = None | Thick liquid – Vocal fold residue at 1 month |
| ns_dac_hatm_1 | 0 = None; 2 = Score 2 | Thick liquid – Subglottic residue at 1 month |
| ns_dac_lenmui_1 | 0 = No; 1 = Yes | Thick liquid – Nasal regurgitation at 1 month |
| ns_dac_km_1 | 0 = None; 1 = Adhered to mucosa as streaks; 2 = Shallow residue; 3 = Deep residue | Thick liquid – Oral cavity residue at 1 month |
| ns_dac_hitsac_1 | 0 = No; 1 = Yes | Thick liquid – Penetration or aspiration at 1 month |
| ns_dac_npn_1 | 0 = No; 1 = Yes | Thick liquid – Use of swallowing maneuver at 1 month |
| ns_dac_tennpn_1 | Text | Thick liquid – Name of effective swallowing maneuver used at 1 month |
| ns_lg_slnt_1 | 0 = No; 1 = Once; 2 = Multiple times | Number of additional swallows with thin liquid at 1 month |
| ns_lg_hm_1 | 0 = None; 1 = Adhered to mucosa as streaks; 2 = Shallow residue; 3 = Deep residue | Thin liquid – Oral cavity residue at 1 month |
| ns_lg_hh_1 | 0 = None; 1 = Adhered to mucosa as streaks; 2 = Shallow residue | Thin liquid – Hypopharyngeal residue at 1 month |
| ns_lg_naptt_1 | 0 = None; 1 = Present; 2 = Score 2 | Thin liquid – Epiglottic residue at 1 month |
| ns_lg_tiendinh_1 | 0 = None; 1 = Present; 2 = Score 2 | Thin liquid – Laryngeal vestibule residue at 1 month |
| ns_lg_daythanh_1 | 0 = None; 1 = Present; 2 = Score 2 | Thin liquid – Vocal fold residue at 1 month |
| ns_lg_hatm_1 | 0 = None; 1 = Present; 2 = Score 2 | Thin liquid – Subglottic residue at 1 month |
| ns_lg_lenmui_1 | 0 = No; 1 = Yes | Thin liquid – Nasal regurgitation at 1 month |
| ns_lg_km_1 | 0 = None; 1 = Adhered to mucosa as streaks; 2 = Shallow residue | Thin liquid – Oral cavity residue at 1 month |
| ns_lg_hitsac_1 | 0 = No; 1 = Yes | Thin liquid – Penetration or aspiration at 1 month |
| ns_lg_npn_1 | 0 = No swallowing maneuver used; 1 = Swallowing maneuver used | Thin liquid – Use of swallowing maneuver at 1 month |
| ns_lg_tennpn_1 | Text | Thin liquid – Name of effective swallowing maneuver used at 1 month |
| sudungnpn_1 | 0 = No; 1 = Yes | Use of swallowing maneuver at 1 month |
| fois_1 | 2 = 2; 5 = 5; 6 = 6; 7 = 7 | FOIS score at 1 month |
| w_3 | Numeric (N = 89; from: 35 to 74) | Weight at 3 months |
| m_3 | 2 = Mainly tube feeding with small oral intake; 3 = Can eat any food if appropriately prepared; 4 = Most foods can be swallowed but require caution to avoid aspiration; 5 = Eats all types of food | Feeding method at 3 months |
| t_3 | 1 = Over 50 minutes or impossible; 2 = 35–45 minutes; 3 = 25–35 minutes; 4 = 15–25 minutes; 5 = Under 15 minutes | Average mealtime duration at 3 months |
| f_3 | 2 = Can swallow thickened liquid; 3 = Can eat porridge; 4 = Can eat soft foods such as soft rice, soft vegetables; 5 = Can eat any type of food | Type of food able to eat at 3 months |
| eat_3 | Numeric (N = 89; from: 0 to 36) | EAT-10 score at 3 months |
| eat_gp_3 | 0 = No; 1 = Yes | EAT-10 grouped score at 3 months |
| eat_cau1_3 | 0 = 0; 1 = 1; 2 = 2; 3 = 3; 4 = 4 | Dysphagia caused weight loss – 3 months |
| eat_cau2_3 | 0 = 0; 1 = 1; 2 = 2; 3 = 3; 4 = 4 | Dysphagia prevented comfortable eating/drinking outside – 3 months |
| eat_cau3_3 | 0 = 0; 1 = 1; 2 = 2; 3 = 3 | Effort needed to swallow liquids – 3 months |
| eat_cau4_3 | 0 = 0; 1 = 1; 2 = 2; 3 = 3; 4 = 4 | Effort needed to swallow solid food – 3 months |
| eat_cau5_3 | 0 = 0; 1 = 1; 2 = 2; 3 = 3 | Effort needed to swallow pills – 3 months |
| eat_cau6_3 | 0 = 0; 1 = 1; 2 = 2; 3 = 3; 4 = 4 | Swallowing is painful – 3 months |
| eat_cau7_3 | 0 = 0; 1 = 1; 2 = 2; 3 = 3; 4 = 4 | Difficulty swallowing reduced eating enjoyment – 3 months |
| eat_cau8_3 | 0 = 0; 1 = 1; 2 = 2; 3 = 3; 4 = 4 | Food stuck in throat while swallowing – 3 months |
| eat_cau9_3 | 0 = 0; 1 = 1; 2 = 2; 3 = 3; 4 = 4 | Coughing during eating/drinking – 3 months |
| eat_cau10_3 | 0 = 0; 1 = 1; 2 = 2; 3 = 3 | Fear of swallowing – 3 months |
| ns_daythanh_3 | 0 = Normal vocal cord movement; 1 = Weakness or unilateral vocal cord paralysis; 2 = Bilateral vocal cord weakness or paralysis | Vocal cord status at 3 months |
| ns_udongdich_3 | 0 = None or a few secretion bubbles not bilateral and not deeply pooled; 1 = Deep pooling or bilateral pooling; 2 = Pooling in laryngeal vestibule disappearing after swallow or cough | Pharyngeal secretion retention at 3 months |
| ns_tinhchatdich_3 | 0 = No retained secretions; 1 = Bubbles; 2 = Mucus; 3 = Bubbles and mucus | Characteristics of retained pharyngeal secretions at 3 months |
| ns_dv_slnt_3 | 0 = No; 1 = Once; 2 = Multiple times | Number of additional swallows with moderately thick liquid at 3 months |
| ns_dv_hm_3 | 0 = None; 1 = Adhered to mucosa as streaks; 2 = Shallow residue | Moderately thick liquid – Oral cavity residue at 3 months |
| ns_dv_hh_3 | 0 = None; 1 = Adhered to mucosa as streaks; 2 = Shallow residue | Moderately thick liquid – Hypopharyngeal residue at 3 months |
| ns_dv_naptt_3 | 0 = None; 1 = Present; 2 = Score 2 | Moderately thick liquid – Epiglottic residue at 3 months |
| ns_dv_tiendinh_3 | 0 = None; 1 = Present | Moderately thick liquid – Laryngeal vestibule residue at 3 months |
| ns_dv_daythanh_3 | 0 = None; 1 = Present | Moderately thick liquid – Vocal fold residue at 3 months |
| ns_dv_hatm_3 | 0 = None | Moderately thick liquid – Subglottic residue at 3 months |
| ns_dv_lenmui_3 | 0 = No; 1 = Yes | Moderately thick liquid – Nasal regurgitation at 3 months |
| ns_dv_km_3 | 0 = None; 1 = Adhered to mucosa as streaks; 2 = Shallow residue; 3 = Deep residue | Moderately thick liquid – Oral cavity residue at 3 months |
| ns_dv_hitsac_3 | 0 = No; 1 = Yes | Moderately thick liquid – Penetration or aspiration at 3 months |
| ns_dv_npn_3 | 0 = No; 1 = Yes | Moderately thick liquid – Use of swallowing maneuver at 3 months |
| ns_dv_tennpn_3 | Text | Moderately thick liquid – Name of effective swallowing maneuver used at 3 months |
| ns_dac_slnt_3 | 0 = No; 1 = Once; 2 = Multiple times | Number of additional swallows with thick liquid at 3 months |
| ns_dac_hm_3 | 0 = None; 1 = Adhered to mucosa as streaks; 2 = Shallow residue; 3 = Deep residue | Thick liquid – Oral cavity residue at 3 months |
| ns_dac_hh_3 | 0 = None; 1 = Adhered to mucosa as streaks; 2 = Shallow residue; 3 = Deep residue | Thick liquid – Hypopharyngeal residue at 3 months |
| ns_dac_naptt_3 | 0 = None; 1 = Present; 2 = Score 2 | Thick liquid – Epiglottic residue at 3 months |
| ns_dac_tiendinh_3 | 0 = None; 1 = Present; 2 = Score 2 | Thick liquid – Laryngeal vestibule residue at 3 months |
| ns_dac_daythanh_3 | 0 = None | Thick liquid – Vocal fold residue at 3 months |
| ns_dac_hatm_3 | 0 = None | Thick liquid – Subglottic residue at 3 months |
| ns_dac_lenmui_3 | 0 = No; 1 = Yes | Thick liquid – Nasal regurgitation at 3 months |
| ns_dac_km_3 | 0 = None; 1 = Adhering to mucosa in streaks; 2 = Shallow residue; 3 = Deep residue | Thick liquid – Oral cavity residue at 3 months |
| ns_dac_hitsac_3 | 0 = No; 1 = Yes | Thick liquid – Penetration or aspiration at 3 months |
| ns_dac_npn_3 | 0 = No; 1 = Yes | Thick liquid – Use of swallowing maneuver at 3 months |
| ns_dac_tennpn_3 | Text | Thick liquid – Name of effective swallowing maneuver at 3 months |
| ns_lg_slnt_3 | 0 = No; 1 = Once; 2 = Multiple times | Number of additional swallows with thin liquid at 3 months |
| ns_lg_hm_3 | 0 = None; 1 = Adhering to mucosa in streaks; 2 = Shallow residue | Thin liquid – Oral cavity residue at 3 months |
| ns_lg_hh_3 | 0 = None; 1 = Adhering to mucosa in streaks; 2 = Shallow residue | Thin liquid – Hypopharyngeal residue at 3 months |
| ns_lg_naptt_3 | 0 = None; 1 = Yes | Thin liquid – Epiglottic vallecula residue at 3 months |
| ns_lg_tiendinh_3 | 0 = None; 1 = Yes; 2 = 2 | Thin liquid – Laryngeal vestibule residue at 3 months |
| ns_lg_daythanh_3 | 0 = None; 1 = Yes; 2 = 2 | Thin liquid – Vocal cord residue at 3 months |
| ns_lg_hatm_3 | 0 = None; 1 = Yes | Thin liquid – Subglottic residue at 3 months |
| ns_lg_lenmui_3 | 0 = No; 1 = Yes | Thin liquid – Nasal regurgitation at 3 months |
| ns_lg_km_3 | 0 = None; 1 = Adhering to mucosa in streaks; 2 = Shallow residue | Thin liquid – Oral cavity residue at 3 months |
| ns_lg_hitsac_3 | 0 = No; 1 = Yes | Thin liquid – Penetration or aspiration at 3 months |
| ns_lg_npn_3 | 0 = No swallowing maneuver used; 1 = Swallowing maneuver used | Thin liquid – Use of swallowing maneuver at 3 months |
| ns_lg_tennpn_3 | Text | Thin liquid – Name of effective swallowing maneuver at 3 months |
| sudungnpn_3 | 0 = No; 1 = Yes | Use of swallowing maneuver at 3 months |
| fois_3 | Numeric | FOIS score at 3 months |
| w_6 | Numeric | Weight at 6 months |
| m_6 | 3 = Able to eat any food if prepared in appropriate form; 4 = Most foods can be swallowed but need caution to avoid aspiration; 5 = Able to eat all kinds of food | Feeding method at 6 months |
| t_6 | 1 = More than 50 minutes or unable; 2 = 35–45 minutes; 3 = 25–35 minutes; 4 = 15–25 minutes; 5 = Less than 15 minutes | Average meal duration at 6 months |
| f_6 | 3 = Able to eat porridge; 4 = Able to eat soft foods like soft rice, soft vegetables; 5 = Able to eat any type of food | Food types able to eat at 6 months |
| eat_6 | Numeric (N = 89; from 0 to 27) | EAT-10 score at 6 months |
| eat_gp_6 | 0 = No; 1 = Yes | EAT-10 score group at 6 months |
| eat_cau1_6 | 0 = 0; 1 = 1; 2 = 2; 3 = 3; 4 = 4 | Dysphagia causes weight loss – 6 months |
| eat_cau2_6 | 0 = 0; 1 = 1; 2 = 2; 3 = 3; 4 = 4 | Dysphagia prevents comfortable eating in public – 6 months |
| eat_cau3_6 | 0 = 0; 1 = 1; 2 = 2; 3 = 3 | Effort needed to swallow liquids – 6 months |
| eat_cau4_6 | 0 = 0; 1 = 1; 2 = 2; 3 = 3 | Effort needed to swallow solids – 6 months |
| eat_cau5_6 | 0 = 0; 1 = 1; 2 = 2; 3 = 3 | Effort needed to swallow pills – 6 months |
| eat_cau6_6 | 0 = 0; 1 = 1; 2 = 2; 3 = 3 | Swallowing is painful – 6 months |
| eat_cau7_6 | 0 = 0; 1 = 1; 2 = 2; 3 = 3 | Difficulty swallowing reduces enjoyment of eating – 6 months |
| eat_cau8_6 | 0 = 0; 1 = 1; 2 = 2 | Food gets stuck in throat when swallowing – 6 months |
| eat_cau9_6 | 0 = 0; 1 = 1; 2 = 2; 3 = 3 | Coughing during meals – 6 months |
| eat_cau10_6 | 0 = 0; 1 = 1; 2 = 2; 3 = 3 | Fear of swallowing – 6 months |
| ns_daythanh_6 | 0 = Normal vocal cord movement; 1 = Weakness or unilateral vocal cord paralysis; 2 = Weakness or bilateral vocal cord paralysis | Vocal cord status at 6 months |
| ns_udongdich_6 | 0 = No or few secretion bubbles not bilateral and not deeply pooled; 1 = Deep pooling or bilateral pooling; 2 = Pooling in laryngeal vestibule disappearing after swallow or cough; 3 = Pooling in laryngeal vestibule not disappearing after swallow or cough | Pharyngeal secretion retention at 6 months |
| ns_tinhchatdich_6 | 0 = No retained secretions; 1 = Bubbles; 2 = Mucus; 3 = Bubbles and mucus | Characteristics of retained pharyngeal secretions at 6 months |
| ns_dv_slnt_6 | 0 = None; 1 = Once; 2 = Multiple times | Number of additional swallows with mildly thick liquids at 6 months |
| ns_dv_hm_6 | 0 = None; 1 = Adherent streaks on mucosa; 2 = Mild residue; 3 = Severe residue | Mildly thick liquid – Oral residue at 6 months |
| ns_dv_hh_6 | 0 = None; 1 = Adherent streaks on mucosa; 2 = Mild residue | Mildly thick liquid – Hypopharyngeal residue at 6 months |
| ns_dv_naptt_6 | 0 = None; 1 = Yes; 2 = 2 | Mildly thick liquid – Epiglottic residue at 6 months |
| ns_dv_tiendinh_6 | 0 = None; 1 = Yes | Mildly thick liquid – Laryngeal vestibule residue at 6 months |
| ns_dv_daythanh_6 | 0 = None; 1 = Yes | Mildly thick liquid – Vocal cord residue at 6 months |
| ns_dv_hatm_6 | 0 = None | Mildly thick liquid – Subglottic residue at 6 months |
| ns_dv_lenmui_6 | 0 = No; 1 = Yes | Mildly thick liquid – Nasal regurgitation at 6 months |
| ns_dv_km_6 | 0 = None; 1 = Adherent streaks on mucosa; 2 = Mild residue | Mildly thick liquid – Oral cavity residue at 6 months |
| ns_dv_hitsac_6 | 0 = No; 1 = Yes | Mildly thick liquid – Penetration or aspiration at 6 months |
| ns_dv_npn_6 | 0 = No; 1 = Yes | Mildly thick liquid – Compensatory swallowing maneuver used at 6 months |
| ns_dv_tennpn_6 | Text | Mildly thick liquid – Name of effective compensatory swallowing maneuver |
| ns_dac_slnt_6 | 0 = None; 1 = Once; 2 = Multiple times | Number of additional swallows with thick liquids at 6 months |
| ns_dac_hm_6 | 0 = None; 1 = Adherent streaks on mucosa; 2 = Mild residue; 3 = Severe residue | Thick liquid – Oral residue at 6 months |
| ns_dac_hh_6 | 0 = None; 1 = Adherent streaks on mucosa; 2 = Mild residue; 3 = Severe residue | Thick liquid – Hypopharyngeal residue at 6 months |
| ns_dac_naptt_6 | 0 = None; 1 = Yes; 2 = 2 | Thick liquid – Epiglottic residue at 6 months |
| ns_dac_tiendinh_6 | 0 = None | Thick liquid – Laryngeal vestibule residue at 6 months |
| ns_dac_daythanh_6 | 0 = None | Thick liquid – Vocal cord residue at 6 months |
| ns_dac_hatm_6 | 0 = None | Thick liquid – Subglottic residue at 6 months |
| ns_dac_lenmui_6 | 0 = No; 1 = Yes | Thick liquid – Nasal regurgitation at 6 months |
| ns_dac_km_6 | 0 = None; 1 = Adherent streaks on mucosa; 2 = Mild residue | Thick liquid – Oral cavity residue at 6 months |
| ns_dac_hitsac_6 | 0 = No | Thick liquid – Penetration or aspiration at 6 months |
| ns_dac_npn_6 | 0 = No; 1 = Yes | Thick liquid – Compensatory swallowing maneuver used at 6 months |
| ns_dac_tennpn_6 | Text | Thick liquid – Name of effective compensatory swallowing maneuver |
| ns_lg_slnt_6 | 0 = None; 1 = Once; 2 = Multiple times | Number of additional swallows with thin liquids at 6 months |
| ns_lg_hm_6 | 0 = None; 1 = Adherent streaks on mucosa; 2 = Mild residue | Thin liquid – Oral residue at 6 months |
| ns_lg_hh_6 | 0 = None; 1 = Adherent streaks on mucosa; 2 = Mild residue | Thin liquid – Hypopharyngeal residue at 6 months |
| ns_lg_naptt_6 | 0 = None; 1 = Yes | Thin liquid – Epiglottic residue at 6 months |
| ns_lg_tiendinh_6 | 0 = None; 1 = Yes | Thin liquid – Laryngeal vestibule residue at 6 months |
| ns_lg_daythanh_6 | 0 = None; 1 = Yes | Thin liquid – Vocal cord residue at 6 months |
| ns_lg_hatm_6 | 0 = None; 1 = Yes | Thin liquid – Subglottic residue at 6 months |
| ns_lg_lenmui_6 | 0 = No; 1 = Yes | Thin liquid – Nasal regurgitation at 6 months |
| ns_lg_km_6 | 0 = None; 1 = Adherent streaks on mucosa; 2 = Mild residue | Thin liquid – Oral cavity residue at 6 months |
| ns_lg_hitsac_6 | 0 = No; 1 = Yes | Thin liquid – Penetration or aspiration at 6 months |
| ns_lg_npn_6 | 0 = No compensatory maneuver used; 1 = Compensatory maneuver used | Thin liquid – Compensatory swallowing maneuver used at 6 months |
| ns_lg_tennpn_6 | Text | Thin liquid – Name of effective compensatory swallowing maneuver |
| sudungnpn_6 | 0 = No; 1 = Yes | Compensatory swallowing maneuver used at 6 months |
| fois_6 | 5 = 5; 6 = 6; 7 = 7 | FOIS score at 6 months |
| sass_0 | Numeric (N = 89; from: 9 to 15) | SASS score before surgery |
| sass_0gp | 0 = Poor; 1 = Good | SASS score before surgery |
| sass_1 | Numeric (N = 89; from: 8 to 15) | SASS score 1 month after surgery |
| sass_1gp | 0 = Poor; 1 = Good | SASS score 1 month after surgery |
| sass_3 | Numeric (N = 89; from: 6 to 15) | SASS score 3 months after surgery |
| sass_3gp | 0 = Poor; 1 = Good | SASS score 3 months after surgery |
| sass_6 | Numeric (N = 89; from: 7 to 15) | SASS score 6 months after surgery |
| sass_6gp | 0 = Poor; 1 = Good | SASS score 6 months after surgery |
