# Supplementary material for: Postoperative swallowing recovery in oral and oropharyngeal cancer: A prospective analysis of functional changes and adjuvant therapy effects
Source: PLoS One. 2026 Jan 27;21(1):e0341116. doi: 10.1371/journal.pone.0341116 (PMC12843576; doi:10.1371/journal.pone.0341116)
Supplement: Attachment — Submitted filename: Response to reviewers.docx [file pone.0341116.s003.docx]

**RESPONSE TO EDITOR AND REVIEWERS**

Thank you for providing thoughtful and valuable feedback on the manuscript. We have responded to the reviewer’s comments below and have changed the text accordingly.

We hope we have satisfactorily addressed all comments and look forward to hearing from you about this submission.

**Academic Editor:** The article needs a major overhaul.

**Response:** Thank you for this assessment. We acknowledge that the manuscript requires substantial revisions. We have carefully reviewed all the major issues raised by Reviewer #1 and the additional comments from the editor, and have made changes across the manuscript (Introduction, Methods, Results, and Discussion) to address them comprehensively.

**Additional Editor Comments:** There are some major issues in the article which the authors need to address.

**Response:** We have responded to the specific issues in detail under the comments below.

**1:** Please ensure that your manuscript meets PLOS ONE's style requirements, including those for file naming.

**Response:** We have reviewed the PLOS ONE style guidelines and have thoroughly formatted the manuscript to ensure compliance with all requirements.

**2:** Please remove your figures from within your manuscript file, leaving only the individual TIFF/EPS image files, uploaded separately. These will be automatically included in the reviewers' PDF.

**Response:** We have removed all figures (Figures 1, 2, and 3) from the main manuscript text. The figures have now been uploaded as separate, high-resolution files as requested.

**3:** Please include captions for your Supporting Information files at the end of your manuscript, and update any in-text citations to match accordingly.

**Response:** We have reviewed the Supporting Information guidelines. Currently, the manuscript does not contain any supplementary files.

Reviewers' comments:

Reviewer's Responses to Questions

**Comments to the Author**

1. Is the manuscript technically sound, and do the data support the conclusions?

The manuscript must describe a technically sound piece of scientific research with data that supports the conclusions. Experiments must have been conducted rigorously, with appropriate controls, replication, and sample sizes. The conclusions must be drawn appropriately based on the data presented.

Reviewer #1: Yes

Reviewer #2: Yes

**Response:** Thank you.

2. Has the statistical analysis been performed appropriately and rigorously?

Reviewer #1: Yes

Reviewer #2: Yes

**Response:** Thank you.

3. Have the authors made all data underlying the findings in their manuscript fully available?

The [PLOS Data policy](http://www.plosone.org/static/policies.action#sharing) requires authors to make all data underlying the findings described in their manuscript fully available without restriction, with rare exception (please refer to the Data Availability Statement in the manuscript PDF file). The data should be provided as part of the manuscript or its supporting information, or deposited to a public repository. For example, in addition to summary statistics, the data points behind means, medians and variance measures should be available. If there are restrictions on publicly sharing data—e.g. participant privacy or use of data from a third party—those must be specified.

Reviewer #1: Yes

Reviewer #2: Yes

**Response:** Thank you.

4. Is the manuscript presented in an intelligible fashion and written in standard English?

PLOS ONE does not copyedit accepted manuscripts, so the language in submitted articles must be clear, correct, and unambiguous. Any typographical or grammatical errors should be corrected at revision, so please note any specific errors here.

Reviewer #1: Yes

Reviewer #2: Yes

**Response:** Thank you.

5. Review Comments to the Author

**REVIEWER #1**

**Major Issues**

**1:** *No sample size calculation was performed; the rationale for including "all consecutive eligible patients" is acceptable but should be explicitly discussed as a limitation regarding statistical power and generalizability.*

**Response:** Thank you for this important comment. We acknowledge the need to be explicit about our sampling method and its implication for the study’s power.

Rationale in Methods: We confirm that the rationale for enrolling all consecutive eligible patients (a pragmatic sampling approach) is already stated in the Methods section: "*To optimize sample size and enhance representativeness by including a diverse patient population, we did not conduct a formal sample size calculation; rather, all eligible patients encountered within the study timeframe were enrolled.*" **(3rd paragraph, Study Design and Participants, Discussion)**

Discussion of Limitations: We have addressed the second part of your comment by adding a new sentence to the Limitations section to explicitly discuss the constraint of statistical power: "*Second, the study employed a pragmatic consecutive sampling approach rather than a formal sample size calculation, which limits the statistical power for more granular subgroup analyses and may affect the generalizability of the findings.*" **(Last paragraph, Discussion)**

**2:** *The follow-up is restricted to 6 months; late dysphagia beyond this time is clinically important. Authors should acknowledge that long-term swallowing impairment (often >12 months) may differ significantly.*

**Response:** We agree that long-term follow-up is crucial for a complete understanding of dysphagia trajectory. We have revised the Discussion section (Limitations) to clearly acknowledge the 6-month follow-up period as a limitation and noted the clinical importance of long-term (e.g., >12 months) swallowing impairment and late effects. In the revised manuscript, we have now stated:

“*Third, in this study, the follow-up period was restricted to 6 months. While this duration captures the critical phase of acute recovery and the effects of adjuvant therapy, it is important to acknowledge that clinically significant late dysphagia often manifests or progresses beyond 12 months post-treatment. Therefore, additional studies with extended follow-up are warranted to better evaluate long-term functional changes.*” **(Last paragraph, Discussion)**

**3:** *The use of GEE models is appropriate, but more details are needed. Were covariates included in the model? How was missing data (if any) handled?*

**Response:** We thank the reviewer for this valuable comment. We have now expanded the Data Analysis section to provide additional clarification. The GEE model included three main predictors (i.e., time point, adjuvant treatment group, and their interaction term) to evaluate longitudinal changes in swallowing outcomes and the differential effects across treatment modalities. No additional covariates were entered because the number of outcome events was limited, and including multiple variables could reduce model stability. The primary objective was to describe recovery trajectories rather than develop a fully adjusted predictive model. The GEE was implemented using an exchangeable working correlation structure with robust (sandwich) standard errors to ensure valid inference even if the working correlation structure was misspecified. The dataset was organized in a long format, in which each record represents a single observation at a specific follow-up time point (e.g., 1, 3, or 6 months) for each patient. This structure differs from the wide format, where each patient contributes only one row with multiple columns representing repeated measurements.

By using the long-format structure, the GEE model treats each observation as an individual data point while accounting for the within-subject correlation across time. As a result, patients with incomplete follow-up data (e.g., missing one time point) are still included in the analysis based on their available observations. This approach maximizes data utilization, avoids case-wise deletion, and provides unbiased population-level estimates under the assumption that data are missing at random. Therefore, missing data were not expected to bias the estimates, and no imputation was required.

In the revised manuscript, we have now stated:

“*To investigate the effect of adjuvant treatment modalities (none, radiotherapy, chemoradiotherapy) on the trajectory of swallowing outcomes while accounting for repeated measurements within individuals over time, Generalized Estimating Equations (GEE) were employed. The model included three main predictors (i.e., time point, adjuvant treatment group, and their interaction term) to examine longitudinal changes in swallowing outcomes and the differential effects across treatment modalities. No additional covariates were entered into the model because the number of outcome events was limited, and the inclusion of further variables could compromise model stability. Moreover, our primary aim was to characterize recovery trajectories rather than to build a fully adjusted predictive model. The GEE approach, using an exchangeable working correlation structure with robust (sandwich) standard errors, inherently accommodates unbalanced repeated measures and utilizes all available data at each time point. Therefore, missing data were not expected to bias the estimates, and no imputation was performed.*” **(Data analysis, Methods)**

**4:** *Results are presented mostly descriptively; effect sizes (e.g., mean differences with 95% CI) would strengthen the interpretation.*

**Response:** We thank the reviewer for this helpful suggestion. Following conventional reporting standards, Table 1 presents the baseline characteristics of the study population, while Table 2 summarizes the longitudinal descriptive outcomes across follow-up time points. We agree that including effect sizes would strengthen interpretation. Accordingly, Figures 3 and 4 now present the estimated effect sizes derived from the GEE model, expressed as mean score changes for continuous outcomes and predicted probabilities for binary outcomes, each accompanied by 95% confidence intervals. To clarify this addition, we have revised the Data Analysis section, to state:

“*From the fitted GEE models, estimated marginal means (for continuous outcomes) and predicted probabilities (for binary outcomes) together with their 95% confidence intervals were computed to quantify effect sizes for each swallowing outcome across treatment groups and time points.*” **(Data analysis, Methods)**

**5:** *The cohort includes a mix of oral cavity and oropharyngeal cancers with different prognostic and functional implications. The manuscript would benefit from subgroup reporting or, at minimum, a discussion of how tumor subsite and reconstructive procedures might influence swallowing recovery.*

**Response:** While our small sample size limits the power for granular subgroup reporting by tumor subsite and reconstruction type (as also noted in the limitations), we agree that we should acknowledge and discuss this heterogeneity. Thus, we have revised the Discussion section to include an expanded paragraph explicitly discussing how the combination of oral cavity and oropharyngeal cancers, along with variations in surgical defects and reconstruction (e.g., free flap vs. primary closure), is a potential confounder” and likely influences the observed swallowing outcomes. In the revised manuscript, we have now stated:

“*Fourth, a key limitation is the heterogeneous nature of the cohort, combining both oral cavity and oropharyngeal cancers. These subsites have distinct surgical approaches and functional outcomes (e.g., free flap reconstruction vs. primary closure), which are known to significantly influence swallowing recovery. Our current analysis does not provide the statistical power to separately model the longitudinal effects for each specific tumor subsite or reconstruction type, representing a potential confounding factor in the observed trajectory results.*” **(Last paragraph, Discussion)**

We also added a sentence to the limitations stating that a detailed comparison of the individual effects of specific surgical defect characteristics on longitudinal swallowing outcomes could not be performed. We have incorporated the statement in the updated manuscript:

*“Lastly, although we utilized validated objective and subjective swallowing assessments, a direct comparison of the individual effects of specific surgical defect characteristics on the longitudinal trajectories of swallowing outcomes by adjuvant treatment could not be performed in this study.*” **(Last paragraph, Discussion)**

**6:** *The paradoxical finding that SASS mean scores improved while the proportion of "poor SASS" patients increased at 3 months should be better explained, possibly by* subgroup *variability or skewed distribution.*

**Response:** Thank you for highlighting this discrepancy. This is an important detail that requires clarification. We have revised the Results and Discussion sections to provide a better explanation. The mean score change is sensitive to overall minor improvement, while the proportion of "poor SASS" (<10) indicates a shift in a subset of patients into the severely impaired category. We added an explanation in the Discussion (linking the mean score improvement with the transient increase in poor SASS scores) suggesting that the poor SASS score increase at 3 months may be linked to the onset of adjuvant therapy effects (mucositis, pain) which disproportionately impacts a subset of patients, leading to a temporary shift in the distribution towards greater severity. The updated manuscript clarifies this point by stating:

*“We noted a transient and seemingly paradoxical increase in the proportion of patients with 'poor SASS' scores (<10) at the 3-month time point, despite an overall improvement in the mean SASS score. This may be attributed to the heterogeneity of the cohort and the acute effects of adjuvant (chemo)radiotherapy that typically commence or peak around this period, causing temporary, severe impairment in a subset of patients (e.g., due to mucositis or pain), thus disproportionately shifting the distribution towards greater severity*” **(Third paragraph, Discussion)**

**7:** *The clinical relevance of changes in EAT-10 and FOIS should be discussed.*

**Response:** We fully agree that the clinical relevance of the magnitude of change in key scores must be discussed. We have revised the Discussion to include a new paragraph discussing the Minimal Clinically Important Difference (MCID) for EAT-10 (e.g., a change of 2-3 points) and the clinical implications of FOIS changes (e.g., the difference between 5 and 6) to frame the observed changes in a clinically meaningful context. This also addresses a minor comment below. We have added references supporting these clinical thresholds. We have now stated:

*”The observed magnitude of change in EAT-10 scores in the overall cohort (e.g., a decrease from 13.5 at 3 months to 8.3 at 6 months) surpasses the generally accepted Minimal Clinically Important Difference (MCID) for EAT-10, which is often cited as a change of 2-3 points (12, 19). Similarly, the changes observed in FOIS scores, particularly the transition from level 5 (total oral diet with multiple consistencies) at 3 months to level 6 (total oral diet with single restriction) at 6 months, represent a clinically significant step toward less restriction. This interpretation is supported by studies demonstrating that the necessary EAT-10 cut-off points for identifying unsafe swallowing vary dramatically depending on the treatment phase (e.g., from 3 at baseline to 15 post-CRT) (30), and that the EAT-10 has a strong correlation with objective functional intake measured by the FOIS (31).*” **(Third paragraph, Discussion)**

**Minor Issues**

**1:** *Some phrasing could be improved for fluency. Minor grammatical errors and typographical inconsistencies should be corrected.*

**Response:** We have thoroughly re-read and edited the entire manuscript for clarity, fluency, and grammatical errors.

**2:** *Literature review is adequate but could be updated with recent systematic reviews and large cohort studies (2021-2024) focusing on swallowing outcomes post-surgery and CRT. Add references on clinically meaningful thresholds for EAT-10 and FOIS.*

**Response:** We have updated the Introduction and Discussion with several more recent references, including systematic reviews (2021-2024), to ensure the literature review is current. In the revision, we have specifically mentioned:

“*However, the impact of treatment often results in significant long-term functional deficits. To date, limited research has focused on the longitudinal changes in swallowing outcomes, especially when comparing subjective and objective metrics during the acute post-treatment phase. Recent large cohort studies highlight that these functional deficits, notably dysphagia, can worsen and persist for up to five years post-treatment (18), underscoring a critical need to monitor these complex trajectories starting from the early period.*” **(Fourth paragraph, Introduction)**

“*Our finding of a dynamic swallowing trajectory, marked by a transient dip around the 3-month mark corresponding to the peak of treatment toxicities, aligns with observations from other prospective studies on head and neck cancer. A five-year prospective study specifically on oral cancer treatment highlighted that swallowing duration and frequency demonstrate longitudinal changes that often do not return to pre-treatment levels, emphasizing the long-term sequelae. The addition of adjuvant radiotherapy or chemoradiotherapy significantly increases the risk of impaired swallowing function, underscoring the necessity of closely tracking outcomes in these subgroups (18).*” **(Second paragraph, Discussion)**

We have also added references for clinically meaningful thresholds for EAT-10 and FOIS, as addressed in Major Issue 7. In the revision, we have specifically mentioned:

*“The observed magnitude of change in EAT-10 scores in the overall cohort (e.g., a decrease from 13.5 at 3 months to 8.3 at 6 months) surpasses the generally accepted Minimal Clinically Important Difference (MCID) for EAT-10, which is often cited as a change of 2-3 points (12, 19). Similarly, the changes observed in FOIS scores, particularly the transition from level 5 (total oral diet with multiple consistencies) at 3 months to level 6 (total oral diet with single restriction) at 6 months, represent a clinically significant step toward less restriction. This interpretation is supported by studies demonstrating that the necessary EAT-10 cut-off points for identifying unsafe swallowing vary dramatically depending on the treatment phase (e.g., from 3 at baseline to 15 post-CRT) (30), and that the EAT-10 has a strong correlation with objective functional intake measured by the FOIS (31).*” **(Third paragraph, Discussion)**

**3: Abstract Conciseness** *The abstract is informative but somewhat lengthy. It could be more concise, emphasizing key results and clinical implications.*

**Response:** We have reviewed and significantly condensed the Abstract while retaining the key findings and clinical implications, making it more concise and focused. We have also ensured that the title and key takeaways are clearly emphasized.

**REVIEWER #2**

**1:** *Minor suggestion to change "due to failed eligibility" to "did not meet eligibility criteria".*

**Response:** We agree. We have revised the text in the Results section and the Flowchart (Fig 1) to replace "failed eligibility" with "did not meet eligibility criteria". We stated:

*“This study initially enrolled 99 patients with oral cavity/oropharyngeal SCC, with 95 cases proceeding to preoperative evaluation after excluding 4 cases that did not meet eligibility criteria*” **(First paragraph, Results)**

**2:** *Similarly suggest changing "disqualified" to removed from analysis as did not meet eligibility criteria.*

**Response:** We agree. We have revised the text in the Results section and the Flowchart (Fig 1) to replace "disqualified" with "removed from analysis as did not meet eligibility criteria."

**Dear Reviewers. Thank you very much for spending your time reviewing our manuscript. All your comments are very thoughtful and helpful.**
